# Physiological health indexes predict deterioration and mortality in patients with COVID-19: a comparative study

**DOI:** 10.18632/aging.203915

**Published:** 2022-02-25

**Authors:** Irina Strazhesko, Olga Tkacheva, Daria Kashtanova, Mikhail Ivanov, Vladislav Kljashtorny, Antonina Esakova, Maria Karnaushkina, Cassandra Guillemette, Amber Hewett, Véronique Legault, Lilit Maytesian, Maria Litvinova, Alan Cohen, Alexey Moskalev

**Affiliations:** 1 Russian Clinical Research Center for Gerontology, Pirogov Russian National Research Medical University of the Ministry of Healthcare of the Russian Federation, Moscow 129226, Russian Federation; 2 Federal State Autonomous Educational Institution of Higher Education “Peoples’ Friendship University of Russia” (RUDN University), Department of Internal Medicine, Moscow 117198, Russian Federation; 3 PRIMUS Research Group, Department of Family Medicine, University of Sherbrooke, Sherbrooke, Quebec J1H 5N4, Canada; 4 Sechenov First Moscow State Medical University of the Ministry of Health of the Russian Federation (Sechenov University), Moscow 119991, Russian Federation; 5 Research Center on Aging, Sherbrooke, Quebec J1H 4C4, Canada; 6 Research Center of Centre Hospitalier Universitaire de Sherbrooke, Sherbrooke, Quebec J1H 5N4, Canada; 7 Institute of Biology, Komi Science Center of Russian Academy of Sciences, Syktyvkar 167000, Russian Federation; 8 The Loginov Moscow Clinical Scientific Center of Moscow Health Department, Moscow 111123, Russian Federation

**Keywords:** biological age, COVID-19, COVID-19 prognosis, SARS-CoV-2, COVID-19 outcome, pandemic

## Abstract

Old age is a crucial risk factor for severe coronavirus disease 2019 (COVID-19), with serious or fatal outcomes disproportionately affecting older adults compared with the rest of the population. We proposed that the physiological health status and biological age, beyond the chronological age itself, could be the driving trends affecting COVID-19 severity and mortality. A total of 155 participants hospitalized with confirmed COVID-19 aged 26–94 years were recruited for the study. Four different physiological summary indices were calculated: Klemera and Doubal’s biological age, PhenoAge, physiological dysregulation (PD; globally and in specific systems), and integrated albunemia. All of these indices significantly predicted the risk of death (p < 0.01) after adjusting for chronological age and sex. In all models, men were 2.4–4.4-times more likely to die than women. The global PD was shown to be a good predictor of deterioration, with the odds of deterioration increasing by 41.7% per 0.5-unit increase in the global PD. As for death, the odds also increased by 68.3% per 0.5-unit increase in the global PD. Our results are partly attributed to common chronic diseases that aggravate COVID-19, but they also suggest that the underlying physiological state could capture vulnerability to severe COVID-19 and serve as a tool for prognosis that would, in turn, help inpatient management.

## INTRODUCTION

Coronavirus disease 2019 (COVID-19) remains one of the main threats to public health worldwide. Owing to the clinical variability of the COVID-19 disease course, it is important to search for predictors that reliably predict the severity of this disease. The pandemic experience has shown that the greatest risks of COVID-19 severe course and unfavorable outcomes of the disease are age and aging-associated diseases; compared to the 50–60-year age group, the risk of death is 23 times higher for individuals aged > 65 years and 100 times higher for those aged > 85 years. The possible causes of aging-related disparities among severe cases of COVID-19 infection have been widely discussed in the scientific literature [1]. In addition to the most obvious explanation, which is the pronounced comorbidity among elderly patients, a hypothesis regarding the influence of immunosenescence has been proposed [2, 3]. Zhavoronkov et al. posited that aging-associated immunosenescence reduces the ability to protect humans against infection and infection causes biological damage to the body, leading to a loss of homeostasis. These factors lead to the acceleration of the aging processes and the worsening of aging-related diseases. Another significant factor in the high mortality from COVID-19 among the elderly population is the accumulation of functional deficits that occur with increasing age and frailty. It has been shown that frailty syndrome is directly related to mortality [4]. In contrast, it is well known that the rate of aging differs significantly among humans. These differences are vividly represented in both persons with early signs of aging and nonagenarians and centenarians who maintain a good physique for a long time. Thus, there is a need to develop a tool for assessing the clinical and physiological states of a person for a more accurate individual prognosis of the course of COVID-19 infection, which could become a scientific basis for making timely and effective clinical decisions. It is especially important to find and validate those predictors of a severe disease course that could predict the outcome of the disease more effectively than the chronological age.

According to existing data, various calculations for assessing physiological state and biological age can be considered promising predictors of the severity of the course of COVID-19 [5], including measures of the biological age, such as the PhenoAge (PA) and Klemera-Doubal method (KD), integrated albunemia, and physiological dysregulation. In a study by Kuo et al. based on data from the UK Biobank, accelerated aging calculated using the PA 10–14 years before the onset of the COVID-19 pandemic was associated with all-cause mortality in patients with COVID-19 [6]. Differences in the methods used to calculate the physiological states may influence their predictive power. Therefore, to determine the most informative method for assessing physiological state or biological age in relation to the prognosis of COVID-19, it is necessary to conduct comparative studies. In this study, we aimed to assess whether different multivariate metrics of physiological state could predict the outcomes of COVID-19 better than the chronological age.

## MATERIALS AND METHODS

This study included men and women aged ≥ 18 years who were hospitalized in the infectious diseases department of the Hospital for War Veterans No. 3 of Moscow Health Department and diagnosed with COVID-19 by PCR testing. Diagnostics and therapy for COVID-19 were performed in accordance with the guidelines of the Ministry of Health of Russia (“Prevention, diagnosis, and treatment of a new coronavirus infection (COVID-19)), version 5 from August 4, 2020; version 6 from April 28, 2020; and version 7 from March 6, 2020. This study was approved by the Local Ethics Committee of the First Moscow State University, named after I. M. Sechenov (Sechenov University), protocol #19-20 (dated July 2, 2020), and conducted according to the guidelines of the Declaration of Helsinki.

The main purpose of this study was to measure the strength of association between the different types of physical states or biological age and the following outcomes: death, deterioration (transition to a more severe degree according to clinical guidelines), or a combination of these two. Multivariate logistic regression was applied to model the odds ratio (OR) of the outcome using sex, chronological age, and physical state or biological age (with calculators described below) as the predictors. All statistical analyses were performed using Stata version 14 software and R language. A two-sided significance level of 0.05 was used.

Different indices were used to assess the individual physiological states. The biomarkers used are listed in Table 1. First, integrated albumin (IA), a physiological emergent process notably related to inflammation [7], was calculated using the calculator provided by Cohen and the following 14 biomarkers: hemoglobin, hematocrit, MCH, mean corpuscular hemoglobin concentration (MCHC), RBC, RDW, platelets, iron, albumin to globulin ratio, calcium, CRP, alkaline phosphatase, and ALT. Second, the biological age was measured using the KD [5, 8], with eight biomarkers selected based on their availability in the dataset, their independence, and their correlation with the chronological age (r > |0.10|), as suggested by Levine et al. [5]: CRP, albumin, total cholesterol, blood urea nitrogen, RDW, platelets, RBC, and lymphocyte percentage. Third, PA was calculated as described by Levine et al. [9] using the albumin, creatinine, serum glucose, CRP, lymphocyte percentage, mean corpuscular volume (MCV), RDW, alkaline phosphatase, WBC, and chronological age. Finally, we calculated the physiological dysregulation (PD) using the MD, as described elsewhere [10–13]. We selected biomarkers based on their stability in three other cohorts and calculated the PD globally and within two physiological systems:

1. The global PD included 14 biomarkers: MCH, RDW, RBC, platelets, percentage of lymphocytes, WBC, CRP, potassium, sodium, hemoglobin, albumin, ALT, AST, and total protein.
2. The PD in the oxygen transport system included the MCHC, MCV, RDW, RBC, and hemoglobin.
3. The PD in the leukopoiesis system included the percentage of neutrophils, WBC, and percentage of lymphocytes.

**Table 1:** Biomarkers, their mean and standard deviation, measure(s) using the biomarker, and log transformation of biomarkers.

**Biomarker**	**Mean ± SD**	**Measure(s)**	**Log-transformation for normality**
Alanine transaminase (ALT, U/L)	49 ± 62	IA, PD (g)	X
Albumin (g/L)	33.8 ± 5.4	IA, KD, PA, PD (g)	
Albumin-globulin ratio	1.16 ± 0.30	IA	
Alkaline phosphatase (U/L)	223 ± 159	IA, PA	X (IA)
Aspartate transaminase (AST, U/L)	67 ± 80	PD (g)	X
Blood urea nitrogen (BUN) (mmol/L)	8.0 ± 6.0	KD	X
Calcium (mmol/L)	0.90 ± 0.40	IA	
Chronological age (years)	64 ± 15	KD, PA	
C-reactive protein (CRP)	117 ± 89	IA, KD, PA, PD (g)	X
Glucose (mmol/L)	8.0 ± 3.6	PA	
Hematocrit (%)	38.71 ± 5.89	IA	
Hemoglobin (g/L)	129 ± 18	IA, PD (g,o)	
Iron (μmol/L)	8.5 ± 5.7	IA	
Lymphocytes (%)	21 ± 15	KD, PA, PD (g,l)	
Mean corpuscular hemoglobin (MCH) (pg)	30.1 ± 2.6	IA, PD (g)	
Mean corpuscular hemoglobin concentration (MCHC) (g/dL)	33 ± 1	IA, PD (o)	
Mean corpuscular volume (MCV) (fL)	90.5 ± 6.8	PA, PD (o)	
Neutrophils (%)	72 ± 15	PD (l)	
Platelets (10^9^/L)	198 ± 80	IA, KD, PD (g)	X (KD, PD)
Potassium (mmol/L)	3.9 ± 0.8	PD (g)	
Red blood cell count (RBC, 10^6^/μL)	4.32 ± 0.55	IA, KD, PD (g,o)	
Red cell distribution width (RDW) (%)	14.2 ± 3.4	IA, KD, PA, PD (g,o)	X (IA, KD and PD)
Serum creatinine (mg/dL)	1.37 ± 0.96	PA	
Sodium (mmol/L)	139 ± 5	PD (g)	
Total cholesterol (mmol/L)	4.2 ± 1.2	KD	
Total protein (g/L)	64.0 ± 6.5	PD (g)	
White blood cell count (WBC) (10^9^/L)	7.8 ± 4.4	PA, PD (g,l)	X (PD)

Legend:

IA, integrated albunemia; KD, Klemera and Doubal biological age; PA, PhenoAge; PD, physiological dysregulation.

KD, Klemera and Doubal biological age; PA, PhenoAge; PD, physiological dysregulation.

g: biomarker part of the final 14 biomarkers set used for global physiological dysregulation.

l: biomarker used for physiological dysregulation in the leukopoiesis system.

o: biomarker used for physiological dysregulation in the oxygen transport system.

Note: units presented in this table are not necessarily the units in which biomarkers were used in the calculations; the units were adapted to measures (for example, depending on the existing formula, the reference population, etc.).

Due to the small sample size in our study, we used the National Health and Nutrition Examination Survey as a reference population to scale biomarkers and calculate the variance-covariance matrix [12]. The use of an external reference population was cross-validated with that of an Asian population to assess PD stability across races. As PD generally has a log-normal distribution, we used the standardized logarithm of PD (log(PD)/sd(log(PD))). Missing values for iron (67.1%), alkaline phosphatase (59.4%), calcium (2.6%), and alanine aminotransferase (0.65%) were imputed using the mouse function in R (mice package) for the IA and PA calculations. The biomarkers were log-transformed, if needed, to meet the assumptions of normality before the calculation of all measures was performed.

## RESULTS

A total of 155 participants aged between 26 and 94 years from Moscow and hospitalized in the infectious disease department were recruited for this study. All patients were diagnosed with COVID-19 by polymerase chain reaction (PCR) testing and underwent treatment for confirmed COVID-19 from April 14, 2020, to June 10, 2020. Among the included participants, 47% were women (n = 73) and 53% were men (n = 82). The average age of the participants was 64 years. The average biological age calculated using the PA calculator was 75.3 years and that calculated using the KD calculator was 64 years. The other characteristics and more detailed descriptions are presented in Table 2. All other information about the cohort and measured parameters are presented in the Supplementary Data (Supplementary Tables 1–4 and Supplementary Figure 1).

**Table 2:** Descriptive statistics of physiological state, chronological and biological age according to various calculators.

**Parameter**	**Age, years**	**IA, u.**	**KD, years**	**PA, years**	**PD (g), u.**	**PD (o), u.**	**PD (l), u.**
Cohort size, N	155	155	155	146	154	155	155
Mean	64.02	4.54	64.02	75.30	6.08	1.33	1.83
SD	15.24	2,47	17.31	22.75	1.00	1.00	1.00
95% CI	(61.6; 66.44)	(4.15; 4.93)	(61.27; 66.77)	(71.58; 79.03)	(5.92; 6.24)	(1.17; 1.48)	(1.68; 1.99)
Min	26	-2.8	16.1	24.0	2.9	-1.1	-2.3
Max	94	15.5	110.3	123.6	9.6	4.8	5.0
Median	64	4.4	63.0	76.2	6.1	1.2	1.9
Q1	53	2.9	51.9	57.5	5.3	0.7	1.2
Q3	75	5.8	74.3	90.0	6.6	1.8	2.3

Legend:

IA, integrated albumin; KD, Klemera and Doubal biological age; PA, PhenoAge; PD, physiological dysregulation; U, units.

KD, Klemera and Doubal biological age; PA, PhenoAge; PD, physiological dysregulation.

g: biomarker part of the final 14 biomarkers set used for global physiological dysregulation.

l: biomarker used for physiological dysregulation in the leukopoiesis system.

o: biomarker used for physiological dysregulation in the oxygen transport system.

**Figure 1:**
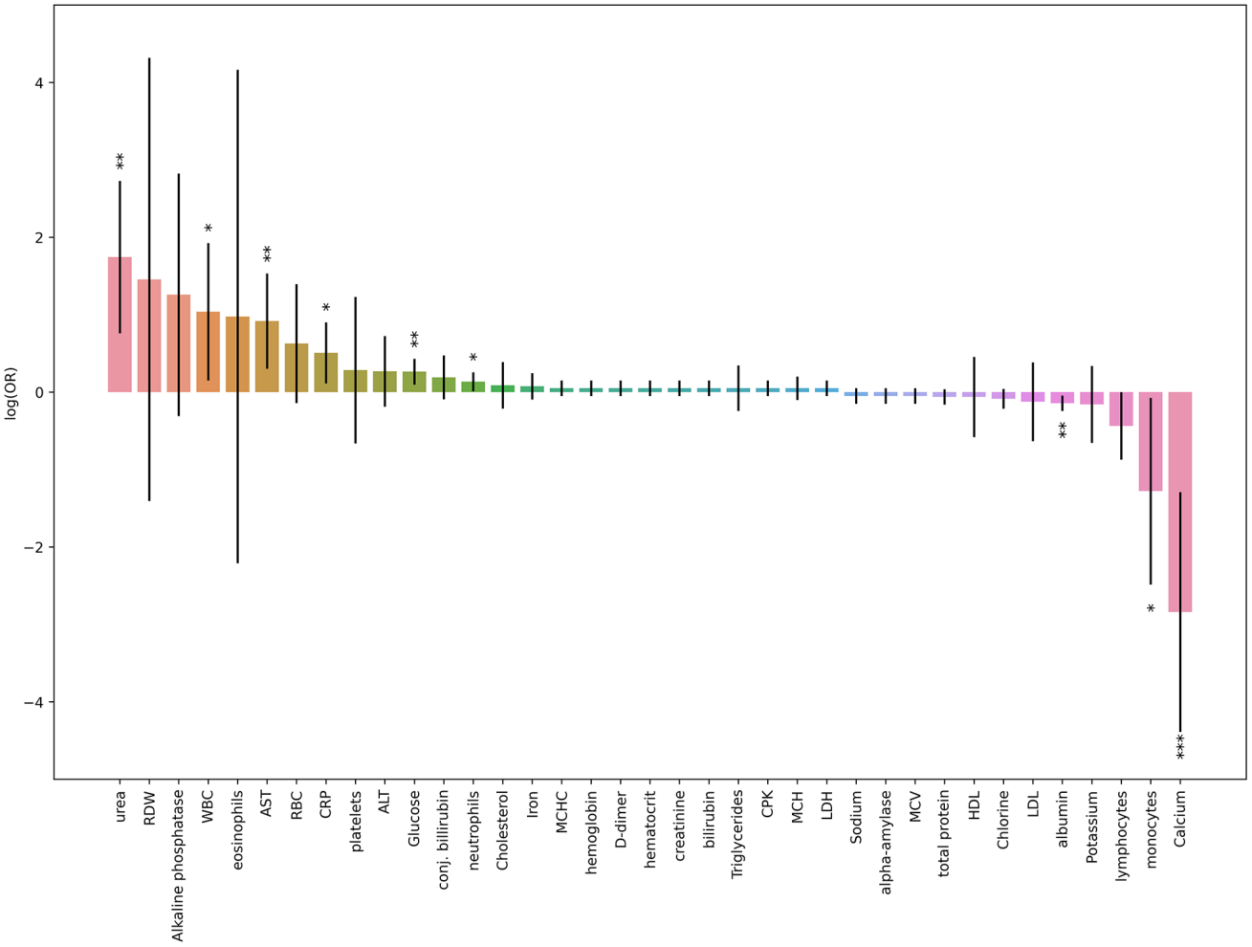
**Results obtained from three-factor logistic regression models for blood tests results parameters and death risk.** Height of each bar depicts log(OR) obtained from logistic regression model (age and sex was taken as covariates), black lines depicts 95% CI for each result. * p-value < 0.05, ** p-value < 0.01, *** p-value < 0.001 (the last one is suitable for Bonferroni adjustment).

**Figure 2:**
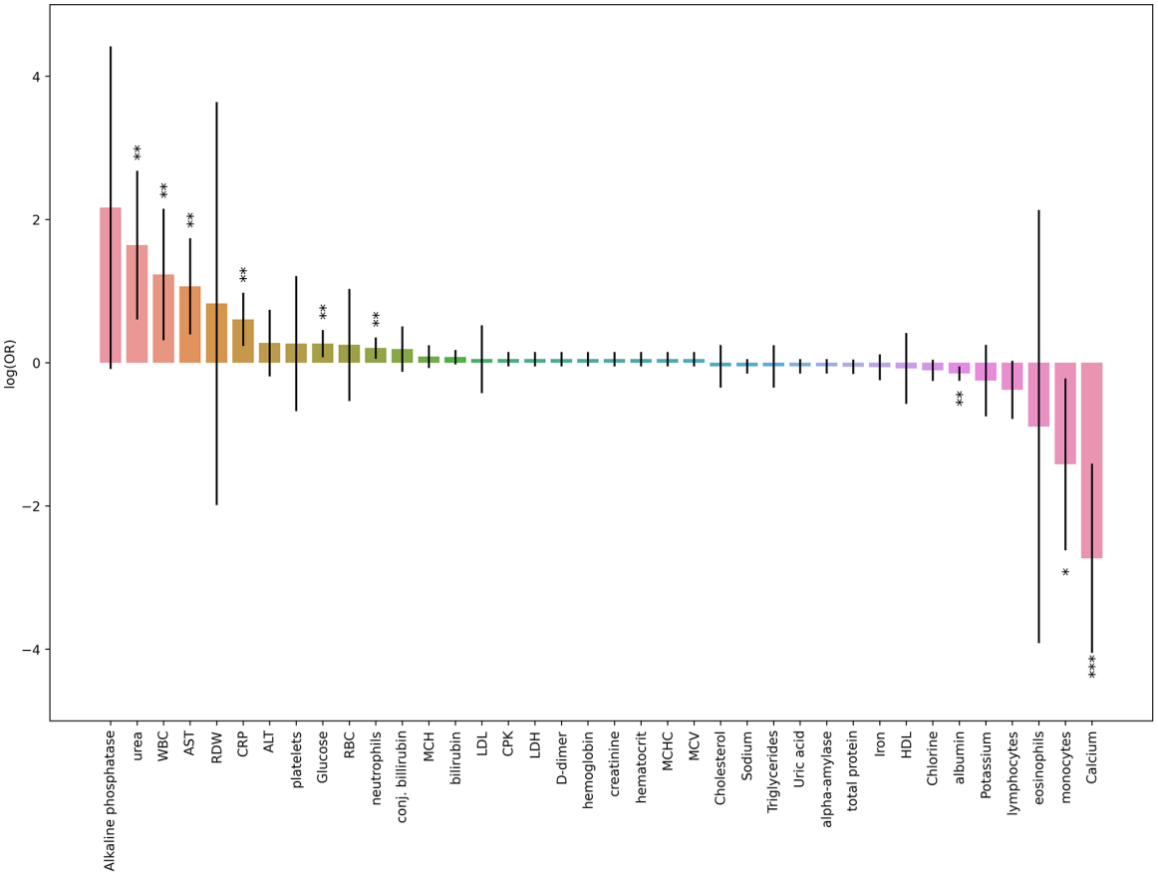
**Results obtained from three-factor logistic regression models for blood tests results parameters and deterioration risk.** Height of each bar depicts log(OR) obtained from logistic regression model (age and sex was taken as covariates), black lines depicts 95% CI for each result. * p-value < 0.05, ** p-value < 0.01, *** p-value < 0.001 (the last one is suitable for Bonferroni adjustment).

First, we performed a three-factor logistic regression analysis with age and sex adjustments to evaluate the association between each cell blood count or biochemical parameters and COVID-19 outcomes (Figures 1, 2).

**Figure 3:**
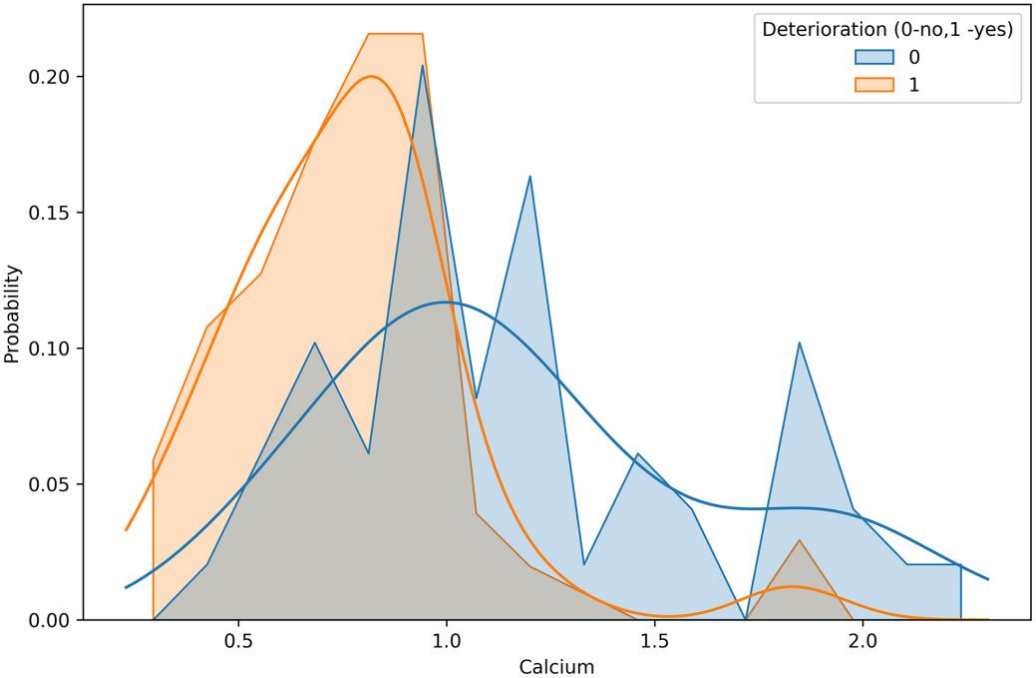
Calcium concentration distributions in groups differed by deterioration outcome.

The most significant association was revealed for calcium level. Low calcium levels were strongly correlated with death and deterioration in patients with COVID-19 (Figure 3). In contrast, the levels of inflammatory markers, urea, liver enzymes, and glucose were increased in the patients with high deterioration and death risks.

**Figure 4:**
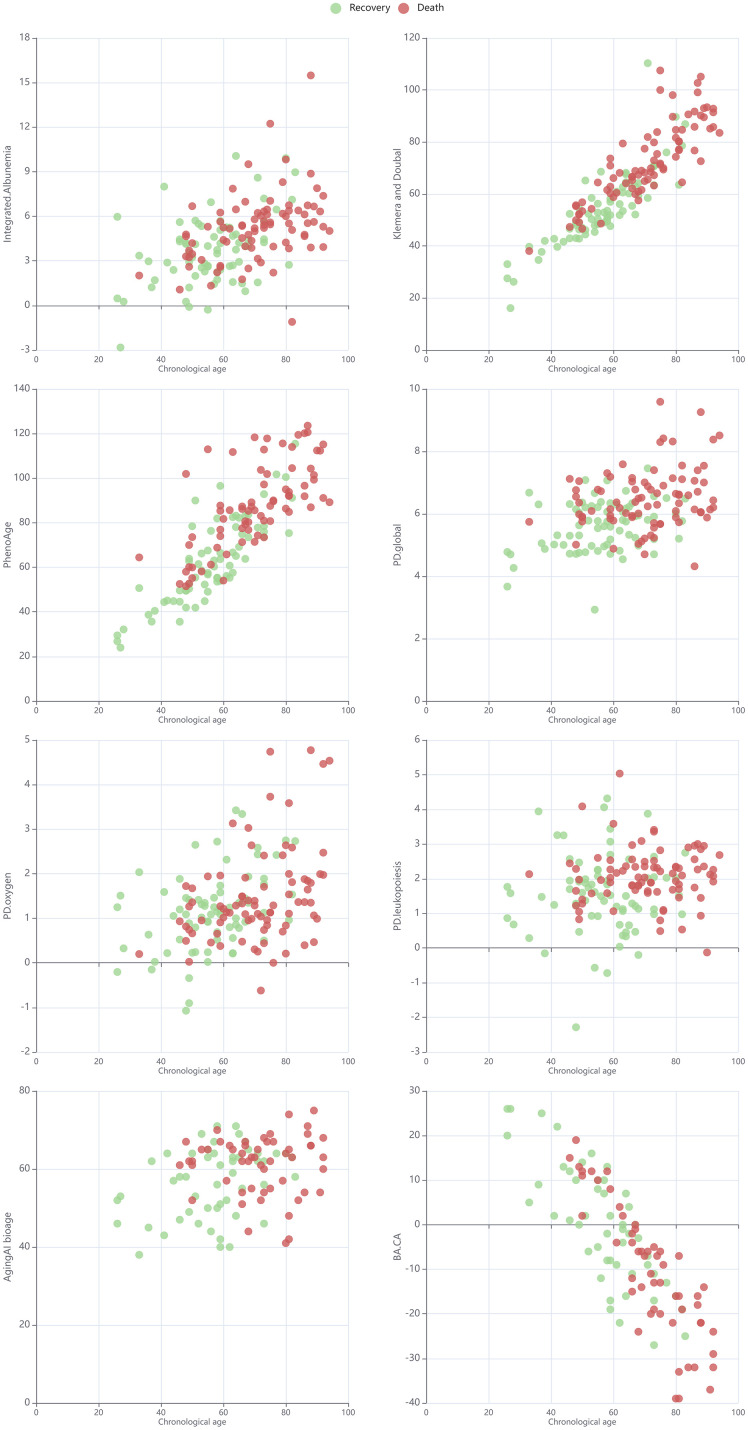
Scatter plot for chronological age and selected metrics of biological age or physiological state in cohorts split by death/recovery.

Analyses using three-factor logistic regression models (Table 3) revealed a significant association between the risk of death and biological age/physiological state based on any of the calculators described above (p < 0.01) after adjusting for chronological age and sex. Thus, the odds of death increased by 68.3% per 0.5-unit increase in the global PD, by 28.5% per 0.5-unit increase in the oxygen transport-PD, by 61.9% per 0.5-unit increase in the leukopoiesis-PD, by 44.9% per 5-unit increase in the KD age, and by 62.3% per 5-unit increase in the PA. In all models, men were 2.4–4.4 times more likely to die than women. The chronological age was not a significant predictor in the KD or PA models (p = 0.429 and p = 0.608, respectively). Across all tests, the integrated albunemia was not associated with deterioration or death (p = 0.52 and p = 0.43, respectively). The dependence between the chronological age and selected metrics of the biological age or physiological state, split by death or recovery, is presented in Figure 4.

**Table 3:** Death OR obtained by multivariate logistic regression.

**Calculator**	**Factor**	**OR**	**p**	**95% CI for OR**	
PD (g) for 0.5 units		1.683	<0.001	1.348	2.101
	Sex (female = ref)	2.553	0.039	1.050	6.209
	Age, for 5 years	1.604	<0.001	1.328	1.937
PD (o)		1.285	0.007	1.069	1.544
	Sex (female = ref)	2.885	0.014	1.237	6.731
	Age, for 5 years	1.575	<0.001	1.313	1.890
PD (l), 0,5 units		1.619	<0.001	1.247	2.101
	Sex (female = ref)	2.378	0.048	1.007	5.617
	Age, for 5 years	1.571	<0.001	1.307	1.887
KD, 5 units		1.449	<0.001	1.177	1.783
	Sex (female = ref)	4.370	0.065	0.915	20.870
	Age, for 5 years	1.147	0.429	0.817	1.609
PA, 5 units		1.623	<0.001	1.247	2.114
	Sex (female = ref)	2.936	0.093	0.835	10.328
	Age, for 5 years	1.079	0.608	0.808	1.440

Legend:

KD, Klemera and Doubal biological age; PA, PhenoAge; PD, physiological dysregulation.

g: biomarker part of the final 14 biomarkers set used for global physiological dysregulation.

l: biomarker used for physiological dysregulation in the leukopoiesis system.

o: biomarker used for physiological dysregulation in the oxygen transport system.

**Figure 5:**
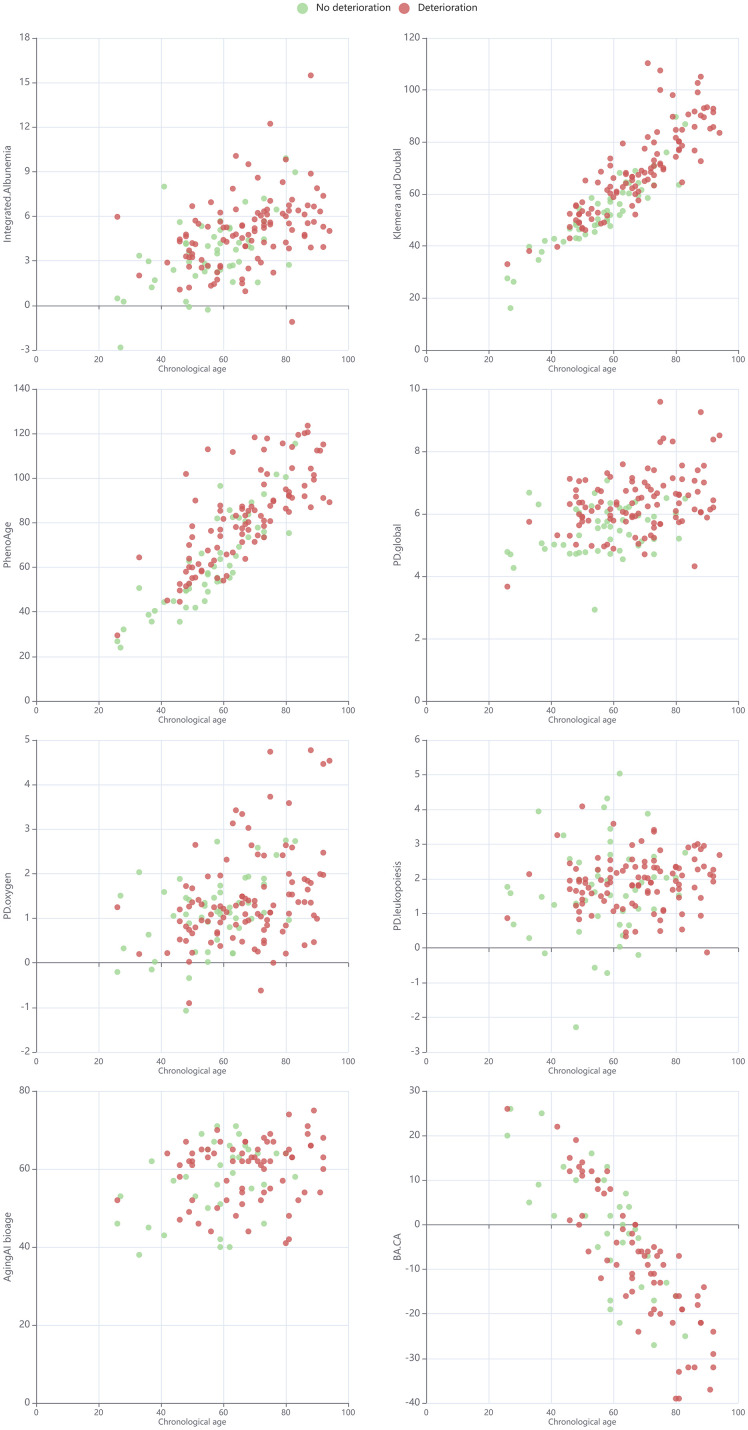
Scatterplot for chronological age and selected metrics of biological age or physiological state in cohorts split by deterioration.

In contrast, the risk of deterioration had no significant association with PD in the oxygen transport system or PA, while the odds of deterioration increased by 41.7% per 0.5-unit increase in the global PD, by 32.9% per 0.5-unit increase in the PD in the leukopoiesis system, and by 20.4% per 5-unit increase in the KD age (Table 4). Except for the model with the PA, in which a significant association was found (p = 0.021), none of the other models showed any statistically significant effect of sex (p > 0.05). Similarly, a weakly significant association of chronological age with the odds of deterioration was revealed only for the model with KD as a predictor (p = 0.010), while, in all other models, chronological age was not statistically significant. The dependence between the chronological age and selected metrics of biological age or physiological state, split by deterioration, is presented in Figure 5.

**Table 4:** COVID-19 course deterioration OR obtained by multivariate logistic regression.

**Calculator**	**Factor**	**OR**	**p**	**95%CI for OR**	
PD (g) for 0.5 units		1.417	<0.001	1.271	1.580
	Sex (female = ref)	1.113	0.593	0.752	1647
	Age, for 5 years	0.988	0.706	0.926	1.05
PD (o)		1.017	0.728	0.927	1.115
	Sex (female = ref)	1.327	0.138	0.913	1.929
	Age, for 5 years	1.031	0.324	0.970	1.097
PD (l), 0,5 units		1.329	<0.001	1.194	1.480
	Sex (female = ref)	1.396	0088	0.951	2.049
	Age, for 5 years	1.011	0.732	0.950	1.076
KD, 5 units		1.204	0.002	1.068	1.358
	Sex (female = ref)	1.583	0.132	0.871	2.877
	Age, for 5 years	0.819	0.010	0.705	0.953
PA, 5 units		1.131	0.116	0.970	1.319
	Sex (female = ref)	2.168	0.021	1.121	4.194
	Age, for 5 years	0.903	0.308	0.742	1.099

Legend:

KD, Klemera and Doubal biological age; PA, PhenoAge; PD, physiological dysregulation.

g: biomarker part of the final 14 biomarkers set used for global physiological dysregulation.

l: biomarker used for physiological dysregulation in the leukopoiesis system.

o: biomarker used for physiological dysregulation in the oxygen transport system.

As for the combined outcome (death or deterioration), the results were very similar to those for the deterioration outcome, which was expected, given that most cases of death involved deterioration (Table 5). The odds of outcome were increased by 41.7% per 0.5-unit increase in the PD global age, by 32.9% per 0.5-unit increase in the PD oxygen age, and by 20.4% per 5-unit increase in the KD age. Sex was not significantly associated with the outcome, while chronological age was significant only in the KD model.

**Table 5:** OR for the combined endpoint (death or deterioration of the patient's condition) obtained by multivariate logistic regression.

**Calculator**	**Factor**	**OR**	**p**	**95% CI for OR**	
PD (g) for 0.5 units		1.417	<0.001	1.271	1.580
	Sex (female = ref)	1.113	0.593	0.752	1.647
	Age, for 5 years	0.988	0.706	0.926	1.054
PD (l), 0,5 units		1.329	<0.001	1.194	1.480
	Sex (female = ref)	1.396	0.088	0.951	2.049
	Age, for 5 years	1.011	0.732	0.950	1.076
KD, 5 units		1.204	0.002	1.068	1.358
	Sex (female = ref)	1.583	0.132	0.871	2.877
	Age, for 5 years	0.819	0.010	0.705	0.953

Legend:

KD, Klemera and Doubal biological age; PA, PhenoAge; PD, physiological dysregulation.

g: biomarker part of the final 14 biomarkers set used for global physiological dysregulation.

l: biomarker used for physiological dysregulation in the leukopoiesis system.

o: biomarker used for physiological dysregulation in the oxygen transport system.

We also checked whether combining these two scales would yield better results. To this end, we built four-factor models including all pairwise combinations of the global PD, PA, and KD for all three outcomes (Tables 6, 7). In all cases, only one of the two metrics showed a significant association with the outcome, whereas the second metric showed no independent contribution. For death, PA and KD remained significant, while global PD was not, and for deterioration, it was global PD that remained significant, while KD and PA did not.

**Table 6:** OR of death obtained by multivariate logistic regression.

**Calculator**	**Factor**	**OR**	**p**	**95% CI for OR**	
	Sex (female = ref)	2.276	0.217	0.616	8.404
	Age, for 5 years	1.069	0.668	0.789	1.448
PD (g) for 0.5 units		1.310	0.160	0.899	1.911
PhenoAge, 5 units		1.541	0.002	1.173	2.024
					
	Sex (female = ref)	3.662	0.125	0.697	19.233
	Age, for 5 years	1.190	0.330	0.838	1.690
PD (g) for 0.5 units		1.352	0.196	0.856	2.138
KD, 5 units		1.324	0.017	1.051	1.667
	Sex (female = ref)	2.907	0.277	0.424	19.940
	Age, for 5 years	1.097	0.684	0.702	1.715
PhenoAge, 5 units		1.146	0.620	0.668	1.967
KD, 5 units		1.368	0.092	0.950	1.971

Legend:

KD, Klemera and Doubal biological age; PA, PhenoAge; PD, physiological dysregulation.

g: biomarker part of the final 14 biomarkers set used for global physiological dysregulation.

l: biomarker used for physiological dysregulation in the leukopoiesis system.

o: biomarker used for physiological dysregulation in the oxygen transport system.

**Table 7:** Odds ratios (OR) of patient deterioration obtained by multivariate logistic regression.

**Calculator**	**Factor**	**OR**	**p**	**95% CI for OR**	
	Sex (female = ref)	1.687	0.140	0.843	3.377
	Age, for 5 years	0.966	0.747	0.784	1.190
PD (g) for 0.5 units		1.479	0.001	1.173	1.864
PhenoAge, 5 units		1.012	0.891	0.854	1.200
					
	Sex (female = ref)	1.011	0.971	0.516	1.984
	Age, for 5 years	0.855	0.049	0.732	0.999
PD (g) for 0.5 units		1.592	<0.001	1.280	1.980
KD, 5 units		1.095	0.154	0.966	1.241
					
	Sex (female = ref)	3.093	0.020	1.194	8.016
	Age, for 5 years	0650	0.009	0.471	0.898
PhenoAge, 5 units		1359	0.061	0.986	1.874
KD, 5 units		1122	0.304	0.901	1.398

Legend:

KD, Klemera and Doubal biological age; PA, PhenoAge; PD, physiological dysregulation.

g: biomarker part of the final 14 biomarkers set used for global physiological dysregulation.

l: biomarker used for physiological dysregulation in the leukopoiesis system.

o: biomarker used for physiological dysregulation in the oxygen transport system.

Thus, we can say that some scales, especially the final 14 biomarker sets used for calculating the global PD, could serve as predictors for both deterioration and death in patients with COVID-19.

## DISCUSSION

Recent studies showed that the severity of COVID-19 was more strongly associated with the biological age rather than the chronological age [14, 15]. In this study, we evaluated the possibility of using physiological state indices to predict disease outcomes. The hypothesis of this study was that summary metrics of physiological state, which take into account morphological, physiological, and functional characteristics of the organism, should better predict disease outcomes. According to our results, some physiological indices predicted a higher risk of mortality and deterioration in the models adjusted for chronological age. The global PD, calculated using the Mahalanobis distance (MD) [11] and including 14 biomarkers (mean corpuscular hemoglobin [MCH], red cell distribution width [RDW], red blood cell [RBC], platelets, percentage of lymphocytes, white blood cell [WBC], C-reactive protein [CRP], potassium, sodium, hemoglobin, albumin, alanine transaminase [ALT], aspartate aminotransferase [AST], and total protein), appeared to be one of the best predictors for death and deterioration of patients with COVID-19. Such results were expected because this calculator consists of crucial parameters for the outcomes. Therefore, WBC, CRP, and other biomarkers, which are commonly used in clinical practice to evaluate COVID-19 severity, along with the chronological age, can be combined into integral models to determine the risk of unfavorable outcomes of the disease. However, the MD calculation involves a non-monotonic manipulation of each component variable and, as such, is not necessarily associated directly with higher levels of individual markers. It is important to note that, unlike other risk scores for COVID-19 severity, this index did not include assessment of comorbidities, for which an assessment could be complicated, especially in case of emergency hospitalizations. Interestingly, integrated albunemia had no association with COVID-19 outcomes, although some of its indicators, including the calcium levels, were strongly correlated with mortality and deterioration. In addition, it should be noted that in the combined indices in the same models, PD did not predict mortality anymore but was still an extremely strong predictor of deterioration. Therefore, we can say that the indices did not measure exactly the same thing.

The aging process is manifested in progressive systemic remodeling of body functioning; therefore, a number of biological dimensions are associated with this process. Most biological indices for age are associated with chronic diseases and unhealthy lifestyle. Strong associations between severe COVID-19 and biological age once again emphasize the importance of preventing aging, both in individuals and in the entire population. The strong association of PD with severe COVID-19 outcomes also suggests the importance of maintaining physiological equilibrium, regardless of age. Unlike PA and KD, the effect of chronological age remained strong in models with PD, suggesting that PD measures information that is more weakly associated with aging and yet is nonetheless critical for health.

Thus, our results are partly attributed to common chronic diseases, which aggravate COVID-19, but also suggest that biological age indices could capture vulnerability to severe COVID-19 and serve as a tool for course prediction and determination of tactics for patient management.

The biological age, as measured by different indices, was associated with a higher risk of mortality and deterioration in the models for which the chronological age and sex were adjusted. Thus, multivariate indices of the physiological state, including the PD, can be used to determine the risk of deterioration and death in a patient. PD measured using the MD could serve as a panel to assess patient risk because it is composed of common markers widely used in clinical practice.

## Supplementary Material

Supplementary Figure 1

Supplementary Tables
