# Supplementary material for: Physiological health indexes predict deterioration and mortality in patients with COVID-19: a comparative study
Source: Aging (Albany NY). 2022 Feb 25;14(4):1611–26. doi: 10.18632/aging.203915 (PMC8908924; doi:10.18632/aging.203915)
Supplement: Supplementary Figure 1 [file aging-14-203915-s001.pdf]

SUPPLEMENTARY FIGURE

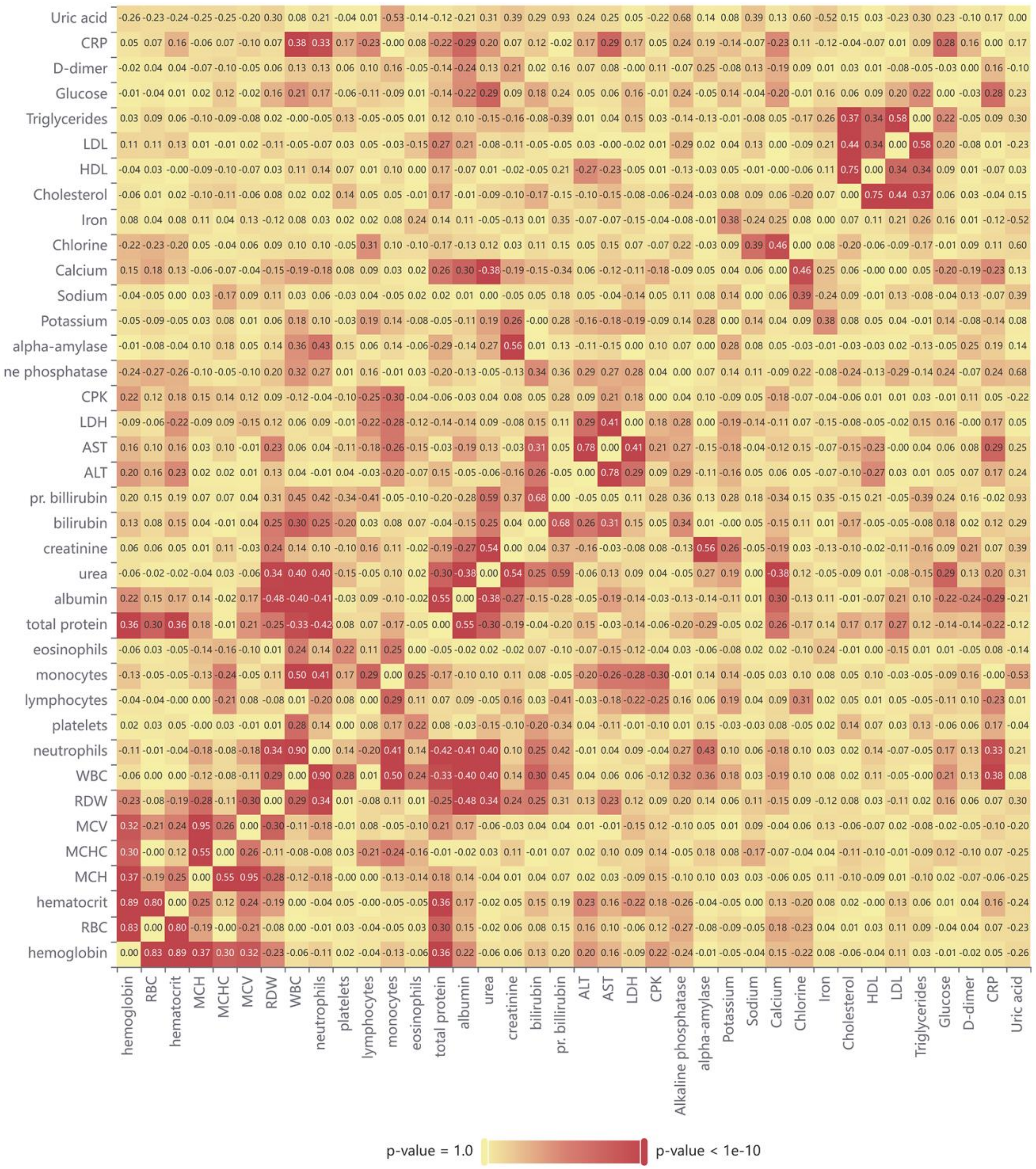

Supplementary Figure 1. Correlation heatmap for CBC indicators, biochemical blood test biomarkers, and additional biomarkers. The color represents the significance of the observed correlation derived from the Pearson's correlation test.
