# Supplementary material for: Physiological health indexes predict deterioration and mortality in patients with COVID-19: a comparative study
Source: Aging (Albany NY). 2022 Feb 25;14(4):1611–26. doi: 10.18632/aging.203915 (PMC8908924; doi:10.18632/aging.203915)
Supplement: Supplementary Tables [file aging-14-203915-s002.pdf]

SUPPLEMENTARY TABLES

Supplementary Table 1. Descriptive statistics of the cell blood count indicators.

Parameter	Hemoglobin	RBC	Hematocrit	MCH	MCHC	MCV	RDW	WBC	Neutrophils	Platelets	Lymphocytes	Monocytes	Eosinophils
N	155	155	155	155	155	155	155	155	155	155	155	155	155
M	129.45	4.32	38.71	30.08	332.40	90.50	14.19	7.75	5.84	197.61	1.31	0.51	0.09
SD	17.67	0.55	5.89	2.62	9.80	6.83	3.36	4.41	4.22	80.23	0.88	0.30	0.11
95% CI	(126.64; 132.25)	(4.23; 4.40)	(37.77; 39.64)	(29.66; 30.50)	(330.84; 333.96)	(89.41; 91.58)	(13.66; 14.72)	(7.05; 8.45)	(5.17; 6.51)	(184.88; 210.34)	(1.17; 1.45)	(0.46; 0.55)	(0.07; 0.10)
Min	43	1.9	14.1	20.9	298.0	65.2	11.4	2.3	1.25	54	0.14	0.09	0
Max	180	5.5	53.5	41.8	362.0	124.4	47.5	28.3	25.2	525	6.9	2.18	0.8
Me	131	4.4	39.0	30.2	331.0	90.6	13.5	6.5	4.46	178	1.2	0.44	0.06
Q1	120	4.0	35.9	28.9	326.0	87.4	12.7	4.9	3.2	143	0.76	0.3	0.034
Q3	140	4.7	42.3	31.4	338.0	93.6	14.8	9.3	7.65	241	1.63	0.63	0.1

Supplementary Table 2. Descriptive statistics of the biochemical blood test indicators.

Parameter	Total protein	Albumin	Urea	Creatinine	Bilirubin	Probillirubin	ALT	AST	LDH	CPK	Alkaline phosphatase
N	155	155	155	155	155	29	154	154	137	124	63
M	63.99	33.76	8.00	120.94	13.00	9.59	49.18	67.08	852.91	316.94	223.27
SD	6.47	5.37	6.02	84.84	9.91	4.95	62.03	79.58	467.86	329.73	158.91
95% CI	(62.97; 65.02)	(32.91; 34.61)	(7.05; 8.96)	(107.48; 134.4)	(11.42; 14.57)	(7.71; 11.47)	(39.30; 59.05)	(54.42; 79.75)	(773.86; 931.96)	(258.33; 375.56)	(183.25; 263.29)
Min	45	18.8	2.1	37.0	4.0	3	3	10	231	12	74
Max	79	46.0	45.3	761.0	69.2	19.9	445	610	2591	1986	1136
Me	64.9	34.0	6.2	99.0	10.4	8.7	32	43	748	197.5	182
Q1	60	30.0	4.4	87.0	7.3	5.2	19	28	506	106.5	137
Q3	68	37.8	8.5	128.0	14.1	13.6	52	66	1039	378.5	241
**Parameter**	**Alphaamylase**	**Potassium**	**Sodium**	**Calcium**	**Chlorine**	**Iron**	**Cholesterol**	**HDL**	**LDL**	**Triglycerides**	**Glucose**
N	55	155	155	151	76	51	155	109	109	154	155
M	59.91	3.86	138.93	0.90	102.18	8.46	4.23	1.52	2.67	2.59	7.96
SD	50.05	0.73	5.12	0.40	5.74	5.69	1.25	0.88	0.89	1.24	3.60
95% CI	(46.38; 73.44)	(3.75; 3.98)	(138.12; 139.74)	(0.83; 0.96)	(100.87; 103.5)	(6.86; 10.06)	(4.03; 4.43)	(1.35; 1.68)	(2.50; 2.83)	(2.40; 2.79)	(7.38; 8.53)
Min	17	2.4	119	0.23	79	2.2	1.3	0.3	0.9	0.61	4.2
Max	222	6.3	160	2.3	121	26.4	7.8	4.5	5.8	6.9	23.6
Me	47	3.8	139	0.86	102	6.2	3.9	1.2	2.7	2.5	6.6
Q1	31	3.3	136	0.63	99	4.1	3.2	0.9	2.1	1.8	5.7
Q3	65	4.3	142	1	106	10.7	5	2	3.1	3.1	8.6

Supplementary Table 3. Descriptive statistics for additional markers.

Parameter	D-dimer	CRP	Uric acid
N	128	155	18
M	1527.77	117.43	262.67
SD	1319.41	88.63	104.66
95% CI	(1297.00; 1758.54)	(103.36; 131.49)	(210.62; 314.71)
Min	76	2	135
Max	4000	436	567
Me	904	102	254
Q1	430	51	194
Q3	2575	162	324

Supplementary Table 4. Descriptive statistics for categorical data.

Factor	Meaning	Number of patients (%)
Sex	Male	82/155 (52.9%)
	Female	73/155 (47.1%)
DM2	No	126/155 (81.3%)
	Yes	29/155 (18.7%)
CAD	No	69/155 (44.5%)
	Yes	86/155 (55.5%)
AH	No	51/155 (32.9%)
	Yes	104/155 (67.1%)
CHF	No	93/155 (60.0%)
	Yes	62/155 (40.0%)
Onco Anamnesis	No	143/155 (92.3%)
	Yes	12/155 (7.7%)
COPD	No	129/155 (832%)
	Yes	26/155 (16.8%)

Note: N, cohort size; M, mean, DM2, diabetes mellitus type 2; CAD, coronary artery disease; AH, arterial hypertension; CHF, chronic heart failure; COPD, chronic obstructive pulmonary disease; CBC, cell blood count.
